# An Unveiling of the Misdiagnosis of Granulomatosis with Polyangiitis as Acute Sinusitis: A Case Report

**DOI:** 10.3390/diagnostics15172218

**Published:** 2025-09-01

**Authors:** Qi Wang, Yi Ling, Yangyiyi Huang, Lijing Zhao, Zhewei Lou, Guokang Fan, Jing Xue

**Affiliations:** 1 Department of Otolaryngology, The Second Affiliated Hospital, Zhejiang University School of Medicine, Hangzhou 310009, China; 2515143@zju.edu.cn (Q.W.); lingyi3972@gmail.com (Y.L.); 2317155@zju.edu.cn (Y.H.); zhaolijing@zju.edu.cn (L.Z.); zheweilou@163.com (Z.L.); 2 Department of Rheumatology, The Second Affiliated Hospital, Zhejiang University School of Medicine, Hangzhou 310009, China

**Keywords:** granulomatosis with polyangiitis, ANCA, early diagnosis, acute sinusitis, otolaryngologic manifestations, imaging features

## Abstract

**Background and Clinical Significance**: Granulomatosis with polyangiitis (GPA), an immune-mediated systemic small-vessel vasculitis affecting the upper/lower respiratory tracts and kidneys, frequently presents with non-specific nasal symptoms that lead to misdiagnosis. **Case Presentation**: We report a case of a 55-year-old female with GPA complicated by Bartter syndrome. She presented with one month of left nasal congestion, rhinorrhea, epistaxis, and headache. Initial diagnosis was acute sinusitis. Computed tomography (CT) revealed left maxillary and ethmoid sinus inflammation with bone destruction, while metagenomic next-generation sequencing (mNGS) suggested conventional bacterial infection. Postoperative pathology demonstrated chronic mucosal inflammation with lymphoid tissue hyperplasia. GPA was ultimately diagnosed based on PR3-ANCA seropositivity and chest CT findings of cavitary pulmonary nodules. Postoperatively, severe hypokalemia and hypomagnesemia secondary to Bartter syndrome emerged. Following electrolyte correction, induction therapy with glucocorticoids and cyclophosphamide was initiated. **Conclusions**: This case underscores that GPA’s head and neck manifestations are frequently misdiagnosed as infections or malignancies. Early diagnosis requires vigilance for GPA ‘red flags’, such as refractory nasal symptoms to conventional therapy (e.g., bloody rhinorrhea), characteristic CT findings (e.g., sinus opacification without ostiomeatal complex obstruction), and nasal endoscopy findings (e.g., ulcers/crusting). Otolaryngologists play a pivotal role in recognizing early disease onset and initiating timely treatment.

## 1. Introduction

Granulomatosis with polyangiitis (GPA), formerly termed Wegener’s granulomatosis, has been classified as an anti-neutrophil cytoplasmic antibody (ANCA)-associated vasculitis (AAV) since 2012 [1]. Pathologically, GPA manifests as a triad of necrosis, granulomatous inflammation, and vasculitis affecting the upper/lower respiratory tract and kidneys. Notably, 85–100% of GPA patients exhibit otolaryngologic symptoms—such as chronic rhinosinusitis or otitis media—which frequently precede systemic involvement by months [2]. These indolent presentations, combined with non-specific radiological findings, create significant diagnostic challenges due to their overlap with common inflammatory sinus diseases. Chronic rhinosinusitis (CRS) affects 5–12% of the global population, with acute cases occurring in 6–15% annually [3]. In contrast, GPA prevalence is markedly lower (~5/100,000 in Europe; ~2/100,000 in East Asia) [4,5], rendering it a rare mimic of CRS. This rarity and symptomatic overlap contribute to frequent diagnostic delays, reported in 60% of AAV patients, with a median diagnostic delay of 6 months [6]. Early recognition is critical: AAV confers a 2.3-fold mortality risk versus the general population [7]. While GPA mortality remains comparable to baseline without renal involvement, kidney disease—often precipitating acute kidney injury—markedly elevates mortality [7]. Consequently, otolaryngologists serve as frontline clinicians encountering initial GPA manifestations. Their ability to distinguish GPA mimicking routine CRS is vital for timely treatment initiation. Nevertheless, awareness of GPA’s sinonasal presentation is still not always sufficient in otolaryngology practice.

Herein, we report a case of GPA complicated by Bartter syndrome initially misdiagnosed as acute sinusitis. This study aims to systematically characterize the otolaryngological manifestations (symptoms, signs, and imaging features) in GPA patients to facilitate early disease detection.

## 2. Report of a Case

A 55-year-old woman presented with a 1-month history of left-sided nasal congestion, rhinorrhea, epistaxis, and headache. Her medical history included Bartter syndrome diagnosed 8 years prior, managed with chronic calcium carbonate and sodium–potassium–magnesium supplementation.

In late November 2024, she developed left nasal congestion, sneezing, blood-tinged purulent rhinorrhea, left-sided facial and cranial distension pain, ocular discomfort, and gingival swelling. Initial evaluation at a local hospital diagnosed acute sinusitis. Treatment with normal saline nasal irrigation, mometasone furoate nasal spray, and oral cefuroxime provided no symptomatic relief, necessitating ongoing acetaminophen analgesia.

**Figure 1:**
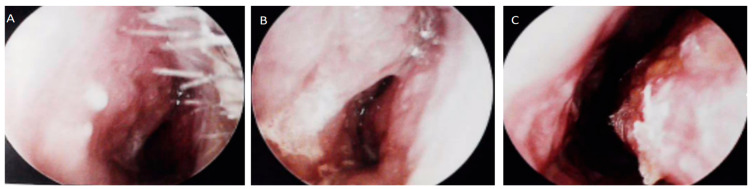
Nasal endoscopy findings. (**A**–**C**) The nasal endoscopy examination revealed that the mucous membranes of the left inferior turbinate and middle turbinate were swollen, with crusts attached, and there was sticky nasal discharge in the common nasal meatus.

**Figure 2:**
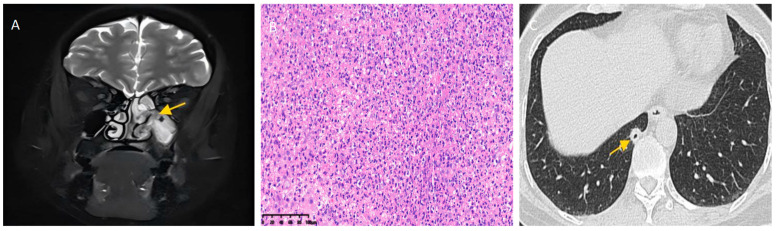
Sinuses magnetic resonance imaging (MRI) and lung computed tomography (CT) findings, pathological results. (**A**) MRI detected sinusitis in the left maxillary and ethmoid sinuses with thickened sinus cavity mucosa indicated by the yellow arrow. (**B**) Hematoxylin–eosin staining, 200×. Pathological results indicated chronic mucosal inflammation and lymphoid tissue hyperplasia, likely reactive hyperplasia dominated by T cell proliferation. (**C**) Lung CT revealed a nodule with cavity formation in the posterior basal segment of the right lower lobe (yellow arrow), suggesting granulomatous lesions first.

**Figure 3:**
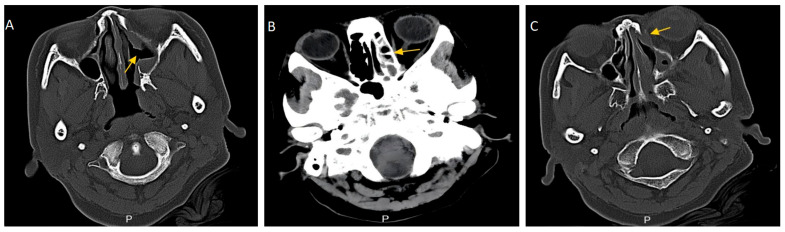
Sinus CT findings. (**A**,**B**) CT scan showed inflammation in the left maxillary sinus and ethmoid sinus (yellow arrow). (**C**) There was bone destruction on the medial wall of the left orbit (yellow arrow).

On 26 December 2024, nasal endoscopy demonstrated mucosal edema of the left inferior and middle turbinates, with minimal crusting and viscous discharge in the common meatus (Figure 1A–C). Subsequent cranial magnetic resonance imaging (MRI) (28 December 2024) revealed left maxillary and ethmoid sinusitis with mucosal thickening (Figure 2A). Sinus computed tomography (CT) (2 January 2025) confirmed inflammatory changes in the left maxillary and ethmoid sinuses, osteolysis of the left medial orbital wall, and significant bony hyperplasia within the ethmoid sinus (Figure 3A–C). Concurrent hematological analysis (2 January 2025) showed marked leukocytosis (WBC 17.6 × 10^9^/L; reference: 3.5–9.5 × 10^9^/L) and neutrophilia (absolute count 14.72 × 10^9^/L, percentage 83.7%). Initial outpatient management with intravenous ceftriaxone (2 g daily) for presumed acute sinusitis failed to produce clinical improvement.

Due to poor response to conservative therapy, preoperative differential diagnoses included “1. Left acute sinusitis (invasive fungal sinusitis to be ruled out), 2. Left nasal lymphoma to be ruled out.” Endoscopic sinus surgery for the left maxillary and ethmoid sinuses was performed on 15 January 2025. Intraoperative findings included diffuse mucosal swelling of the left nasal cavity and septum, swollen maxillary sinus mucosa with cheesy purulent plaques, and hyperplastic, sclerotic ethmoid bone. Frozen section histopathology revealed extensive lymphoid proliferation, suggestive of lymphoma pending definitive histology and immunohistochemistry (IHC). Subsequent metagenomic next-generation sequencing (mNGS) of left nasal mucosa and pus (16 January) detected *Prevotella* spp. and *Porphyromonas endodontalis*.

Postoperatively, intravenous cefoperazone/sulbactam (2 g q8h) and methylprednisolone (40 mg daily, tapered to 8 mg daily over 3 days) were initiated. Headache showed transient improvement. Blood analysis (18 January 2025) showed marked elevation of C-reactive protein (100.3 mg/L). Definitive histopathology (23 January) confirmed chronic mucosal inflammation with lymphoid hyperplasia. Integrated with immunohistochemistry (IHC) and molecular pathology (negative clonal TCR gene rearrangement), findings supported reactive lymphoid hyperplasia characterized by predominant T-cell proliferation (Figure 2B). Cranial MRI and lumbar puncture (24 January) were unremarkable, including normal cerebrospinal fluid (CSF) pressure, routine analysis, and biochemistry. Following Infectious Disease consultation, antibiotic therapy was de-escalated to imipenem/cilastatin (0.5 g q8h).

Her underlying Bartter syndrome triggered severe postoperative hypokalemia (2.55 mmol/L ↓) and hypomagnesemia (0.39 mmol/L ↓) on 25 January. Immunosuppressants were temporarily withheld, with initiation of aggressive intravenous magnesium sulfate and high-dose oral potassium chloride supplementation. As infectious and neoplastic etiologies failed to fully explain the clinical course, rheumatology/immunology consultation was sought. Immunological workup on 25 January revealed elevated complement C3 (2.00 g/L ↑) and C1q (388 mg/L ↑), and a positive anti-PR3 IgG antibody (36.1 (++) AU/mL ↑). High-resolution chest CT on 27 January 2025, identified a cavitary nodule in the posterior basal segment of the right lower lobe, suggestive of a granulomatous lesion (Figure 2C). No obvious endoluminal or wall abnormalities of the trachea and main bronchi were detected on this CT scan. Concurrent urinalysis showed no hematuria, proteinuria, or cellular casts.

**Figure 4:**
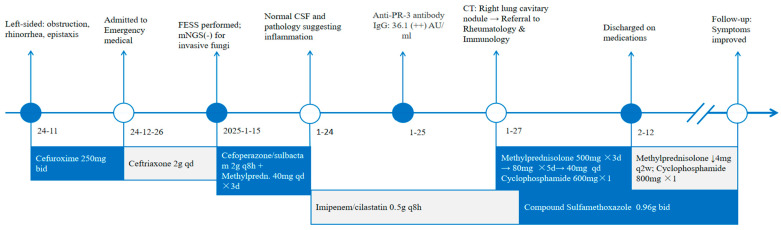
The treatment timeline of the patient. Abbreviations: mNGS, metagenomic next-generation sequencing; FESS, functional endoscopic sinus surgery; CSF, cerebrospinal fluid; CT, computed tomography.

Following correction of electrolyte abnormalities, the patient received methylprednisolone pulse therapy (500 mg/day for 3 days), subsequently tapered to 80 mg/day for 10 days, then 40 mg/day until discharge. Intravenous cyclophosphamide (600 mg) was administered on 8 February. Co-trimoxazole (trimethoprim-sulfamethoxazole, 0.96 g twice daily) was initiated for infection prophylaxis. At discharge, C-reactive protein levels had declined to 15 mg/L. Post-discharge, methylprednisolone was tapered by 4 mg every 2 weeks (starting from 40 mg/day). A subsequent intravenous cyclophosphamide dose (800 mg) was administered on 14 March. At the 2-month follow-up, the patient demonstrated resolution of headache along with improved nasal congestion and rhinorrhea; however, nasal crusting persisted. The treatment timeline is summarized in Figure 4.

## 3. Discussion

GPA predominantly affects individuals aged 40–55 years, with no significant sex predilection [8]. Environmental triggering factors, such as chronic nasal Staphylococcus aureus colonization, silica exposure, and certain drug treatments, are superimposed upon a background of genetic susceptibility [9]. This genetic predisposition involves polymorphisms in the HLA-DPB1 and PRTN3 genes [10]. The typical manifestation of generalized GPA is the triad including upper respiratory tract inflammation, pneumonia, and glomerulonephritis [5]. The main early atypical manifestations include chronic bloody rhinorrhea/crusting, recurrent epistaxis, nasal ulcers, nasal obstruction; when the ears are involved, there may be conductive hearing loss and otorrhea. Crucially, these otorhinolaryngological symptoms are characteristically persistent and refractory to conventional therapies.

Nasal endoscopy in GPA typically reveals mucosal swelling, crusting, and possible scar hyperplasia, whereas CRS primarily demonstrates mucosal edema and polypoid changes with infrequent crusting or ulceration. Sinus CT in GPA shows distinct patterns differing from conventional CRS, invasive fungal sinusitis, and sinonasal malignancies. Its specific signs can be summarized as follows: 1. The sinus mucosa will show different degrees of thickening, and the inside of the sinus is filled with soft tissue shadows, but fluid levels rarely appear. 2. The maxillary sinus is the most severely affected sinus, followed by the ethmoid sinus, sphenoid sinus, and finally the frontal sinus. Maxillary involvement often combines bone destruction with hyperplasia, creating an irregular “double-line” sign and sinus narrowing. 3. The inferior and middle turbinates resorption and potential septal perforation. 4. Absence of polypoid lesions in the ostiomeatal complex (OMC). 5. Unilateral or bilateral involvement [11,12]. In contrast, CT manifestations of CRS involve mucosal thickening, air-fluid levels, the OMC obstruction by polyps and edematous mucosa, and mucoceles. In invasive fungal sinusitis, the acute type is mainly characterized by bone destruction of the paranasal sinuses and thickening of the mucosa, while in the chronic type, there are sinus wall sclerosis, bone destruction and thickening of the mucosa [13]. Malignant bone destruction is infiltrative, margin-irregular, and non-hyperplastic, often with exophytic neoplasms. Squamous cell carcinoma presents as an irregular mass with extensive bone destruction; adenocarcinoma appears as a soft-tissue mass with occasional calcification; lymphoma shows diffuse infiltrative growth with mild bone destruction [14].

Our patient presented with apparent left maxillary sinusitis. Intraoperatively, however, the maxillary sinus ostium was patent and the OMC unobstructed. While conventional sinusitis typically involves ostial obstruction causing drainage impairment and secondary infection, GPA-related sinusitis stems from systemic vasculitis rather than mechanical blockage. Instead, GPA sinusitis is driven by extensive mucosal/submucosal inflammation, explaining the absence of fluid levels on CT. Pathologically, GPA sinusitis features Th1/Th17 inflammation with neutrophil infiltration, vasculitis, and necrotizing granulomas causing tissue destruction and fibrosis, contrasting with the Th2-mediated mucosal edema and hyperplasia seen in ordinary sinusitis [15,16].

Tateyama et al. [17] reported that the CT manifestations of GPA included mucosal thickening in 94.1%, bony thickening in 70.6%, bone destruction in 23.5%, and intraorbital invasive masses in 17.6% of cases. The average time from symptom onset to initial diagnosis in their cohort was 31 months. Among three early-stage cases, two exhibited bony thickening and one showed bone destruction. A review by D’Anza et al. [12] indicated that CT findings in GPA consisted of mucosal thickening in 87.7%, bone destruction in 59.9%, and nasal septal erosion in 59.4% of cases. However, since the timing of imaging relative to disease course was not specified, correlation with disease severity could not be established. A review by Coordes et al. [18] states that a septal perforation becomes apparent when the diseased tissue is resorbed during disease remission. Recognizing GPA-specific CT features—particularly patent OMC despite sinus opacification—enhances early diagnosis. Advanced signs like the “double-line” sign (bone destruction with hyperostosis), turbinate resorption, and septal perforation typically represent later disease stages and are unreliable early indicators.

The classic pathological triad of GPA is necrosis, granulomatous inflammation, and vasculitis. Central to granulomatous lesions is fibrinoid necrosis of small vessels, surrounded by lymphocytic/monocytic infiltration with epithelioid cells, multinucleated giant cells, and fibroblast proliferation [19]. However, unlike lung biopsies, nasal biopsies for diagnosing GPA rarely show the typical manifestations of necrotizing granulomas with giant cells and neutrophil-dominated vasculitis. In the data reported by Thornton, only 16% of head and neck biopsy specimens could observe the classic pathological triad [20]. Our pathological results also only found non-specific inflammation. Obtaining pathological evidence of GPA in the head and neck requires multiple biopsies. Therefore, we believe that recognizing the symptoms and signs of GPA affecting the upper respiratory tract and the characteristics of imaging are more helpful for early diagnosis than biopsy. Given that CT is difficult to distinguish malignant lesions, histopathological examination is still recommended. It may not always confirm GPA, but can rule out malignant lesions.

The diagnosis of GPA integrates clinical features, histopathology, and ANCA serology. Crucially, while ANCA testing supports diagnosis, Lutalo et al. pointed out that if the clinical and histological examination results point to the diagnosis of GPA, a positive ANCA serology is not a necessary condition for diagnosing GPA [21]. The typical ANCA pattern associated with GPA is c-ANCA that recognizes the autoantigen proteinase 3 (PR3), which is a protease existing in neutrophil granules [22]. In our case, PR3 was also positive. In GPA patients, nearly 20% have perinuclear ANCA that recognizes the autoantigen myeloperoxidase (MPO) [2]. In our case, both C3 and C1q levels were elevated; however, these markers are not specifically associated with the core pathological mechanisms of GPA. They serve only as supporting indicators of the patient’s inflammatory activity.

**Figure 5:**
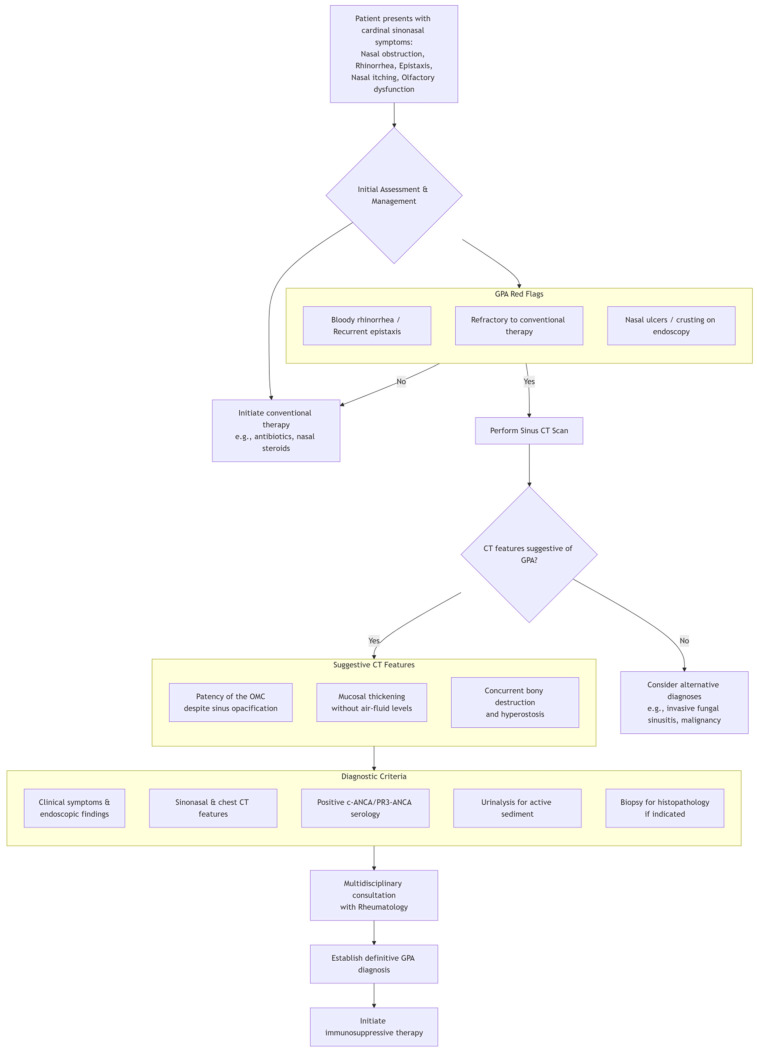
A diagnostic flowchart for GPA with sinusitis as the initial symptom.

In the diagnostic evaluation of GPA presenting with otorhinolaryngological symptoms, vigilance for “GPA red flags” is critical, such as persistent bloody rhinorrhea or recurrent epistaxis, nasal ulcers/crusting, and sinus symptoms refractory to conventional therapy. During imaging examinations, the focus should be placed on evaluating the patency of the OMC despite sinus opacification and mucosal thickening without air-fluid levels. If GPA red flags are identified, further PR3-/MPO-ANCA serology, urinalysis, and nasal biopsy can be arranged. Routine preoperative chest imaging for CRS employs radiography rather than CT due to its lower radiation dose and cost. However, when GPA red flags are present, obtain chest CT to assess pulmonary involvement. GPA may involve the tracheobronchial tree, including subglottic stenosis and ulcerative tracheobronchitis with inflammatory pseudotumors and/or bronchial stenosis. As reported by Terrier et al. [23] tracheobronchial stenosis occurs in 12–23% of patients and may cause life-threatening airway compromise. However, CT may miss early mucosal lesions; bronchoscopy remains the gold standard for definitive evaluation when tracheobronchial involvement is suspected. Figure 5 illustrates the diagnostic workflow for granulomatosis with polyangiitis (GPA) presenting with initial symptoms of sinusitis.

Treatment involves induction therapy (glucocorticoids plus rituximab or cyclophosphamide) followed by maintenance [24]. Rituximab is effective for PR3-ANCA-positive and relapsing disease [25,26], while avacopan reduces steroid use [27].

## 4. Conclusions

Head and neck manifestations of GPA are frequently misdiagnosed as infections or malignancies. Early identification of “GPA red flags” is crucial. Otolaryngologists play a pivotal role in recognizing early disease onset and initiating timely treatment.

## Data Availability

The original contributions presented in this study are included in the article. Further inquiries can be directed to the corresponding author.
